# A Randomized Clinical Trial of an Inactivated Avian Influenza A (H7N7) Vaccine

**DOI:** 10.1371/journal.pone.0049704

**Published:** 2012-12-11

**Authors:** Robert B. Couch, Shital M. Patel, Chianti L. Wade-Bowers, Diane Niño

**Affiliations:** 1 Department of Molecular Virology and Microbiology, Baylor College of Medicine, Houston, Texas, United States of America; 2 Department of Medicine, Baylor College of Medicine, Houston, Texas, United States of America; Instituto Butantan, Brazil

## Abstract

**Background:**

Concern for a pandemic caused by a newly emerged avian influenza A virus has led to clinical trials with candidate vaccines as preparation for such an event. Most trials have involved vaccines for influenza A (H5N1), A (H7N7) or A (H9N2).

**Objective:**

To evaluate dosage-related safety and immunogenicity of an inactivated influenza A (H7N7) vaccine in humans.

**Design:**

One hundred twenty-five healthy young adults were randomized to receive two doses intramuscularly of placebo or 7.5, 15, 45 or 90 µg of HA of an inactivated subunit influenza A (H7N7) vaccine (25 per group), four weeks apart. Reactogenicity was evaluated closely for one week and for any adverse effect for six months after each dose. Serum hemagglutination-inhibiting and neutralizing antibody responses were determined four weeks after each dose and at six months.

**Results:**

Reactogenicity evaluations indicated the vaccinations were well tolerated. Only one subject developed a ≥4-fold serum hemagglutination-inhibition (HAI) antibody response and a final titer of ≥1∶40 four weeks after dose two and only five subjects developed a neutralizing antibody rise and a final titer of ≥1∶40 in tests performed at a central laboratory. Four of the five were given the 45 or 90 µg HA dosage. A more sensitive HAI assay at the study site revealed a dose-response with increasing HA dosage but only 36% in the 90 µg HA group developed a ≥4-fold rise in antibody in this test and only one of these achieved a titer of ≥1∶32.

**Conclusion:**

This inactivated subunit influenza A (H7N7) vaccine was safe but poorly immunogenic in humans.

**Trials Registration:**

ClinicalTrials.gov NCT00546585

## Introduction

The prevailing concept for the origin of new influenza A virus subtypes leading to pandemic influenza in humans is that a new virus with the ability to spread and cause illness contains an avian influenza virus hemagglutinin (HA) and/or neuraminidase glycoprotein (NA) acquired from an avian influenza virus [1]–[3]. This potential exists for the seventeen distinct HAs and nine NAs that have been described among influenza A viruses [1], [4], [5]. The level of concern for this happening was increased when an outbreak with avian influenza A (H5N1) occurred in humans in 1997 in Hong Kong that was from contacts with infected chickens and was reinforced with reappearance of human cases in other parts of the world in succeeding years [6]–[8]. Preparing candidate A/H5N1 vaccines for potential use in humans became an urgent need. Since then, an outbreak with avian influenza A (H7N7) occurred in humans in the Netherlands and cases of avian influenza A (H9N2) have been recognized in humans [9]–[12]. Preparing prototype vaccines for these and perhaps other avian influenza A viruses is now an effort supported by public health authorities so as to be prepared with the knowledge of how to best proceed should one of these subtypes emerge as a pandemic among humans.

The present report is of a report of a clinical trial with an influenza A (H7N7) vaccine prepared by a manufacturer that used established methods for annual production of seasonal influenza vaccine. The objective was to evaluate dosage-related safety and immunogenicity. This vaccine proved to be safe but poorly immunogenic in humans. A second related report is of results comparing a number of the prototype inactivated vaccines containing avian HAs and NAs that have been evaluated in humans, including the A/H7N7 vaccine used in this report, in various in vitro tests in an effort to identify correlates that related to immunogenicity in humans other than the standard single radial immunodiffusion (SRID) assays of HA concentration.

## Materials and Methods

The protocol for this trial and supporting CONSORT checklist are available as supporting information; see Checklist S1 and Protocol S1.

### Subjects and Ethics Statement

Subjects were healthy male and nonpregnant females between the ages of 18 and 40 years. The study was reviewed and approved by the Institutional Review Board for Protection of Human Subjects in research at Baylor College of Medicine before commencing. The study was conducted in a clinic setting and all subjects gave written informed consent before any procedures were conducted. The Declaration of Helsinki principles were followed.

### The H7 Vaccine

The tested vaccine is a monovalent inactivated influenza A (H7N7) vaccine produced by Sanofi Pasteur Inc. using their seasonal vaccine production methods. The virus used was a 6-2 reassortant generated in eggs. The virus donating the HA was A/Mallard/Netherlands/12/2000 (H7N3) and that donating the NA was A/Mallard/Netherlands/2/2000 (H10N7); both are low pathogenic avian influenza viruses. The six internal genes were donated by an influenza A (H1N1) strain, FDA-Resvir-12; the NP gene was from A/Johannesburg/82/96(H1N1) and the other five internal protein genes were from A/Puerto Rico/8/34 (H1N1) [13]. The vaccine virus was grown in embryonated eggs, inactivated with formalin, concentrated and purified and then detergent disrupted with Triton X-100 to produce a subunit virus antigen that was then further purified. It was formulated into single dose vials with 0.05% gelatin but no preservative as dosages of 7.5, 15, and 45 µg per 0.5 ml.

### Sample Size Determination

The sample size (25 per group), was selected by the data coordinating center of the Microbiology Division of the National Institutes of Allergy and Infectious Diseases to provide a robust initial safety database of 125 vaccinees receiving the antigen while providing some information concerning the dose-response immunogenicity in a timely fashion. Immunogenicity results were intended to be sufficient to establish a basis for narrowing the dose selection for further studies in infants, children, and elderly populations. A power calculation was not performed for this Phase I study but for an assumed serious adverse event frequency of 0.1% to 20%, the probability of detecting one or more events for 25 to 100 subjects ranged from 2.47% to 100%.

### Design

The study was conducted in a randomized, double-blind fashion; randomization was conducted after enrollment at the data coordinating center of the sponsor. One hundred thirty-six subjects were screened and 125 were randomly assigned to groups of 25 each to receive 0.5 ml doses intramuscularly of placebo or 7.5 µg, 15 µg, or 45 µg of vaccine HA on days 0 and 28. A fifth group of 25 received one ml of the 45 µg per 0.5 ml dosage (total of 90 µg) at each vaccination. Vaccine dosages were based on single radial immunodiffusion assays (SRID) for HA [14], [15]. Vaccinations were done by persons who did not participate in any further work in the study. Individual vaccine dosages were transmitted to the study site from the coordinating center by code and the site pharmacist, with corroboration by the unblinded vaccination nurse, selected the vaccine dosage syringe to be used. The initial vaccination was on March 10, 2008 and the last on May 19, 2008. The reactogenicity evaluations and antibody assays were performed by persons with no knowledge of the vaccine given. Reactogenicity evaluations consisted of daily recording by subjects of oral temperature, symptoms and any vaccine site changes on a scale of mild, moderate, severe for seven days after each vaccination, a phone check at one to three days and a clinic visit for evaluation at eight to twelve days after each vaccination. Additionally, any adverse effect (AE) up to 28 days after each vaccination and any severe adverse effects (SAE) up to day 208 was recorded and evaluated. Blood for antibody assays was obtained before each vaccination (0 and 28) and on days 56 and 208 after the initial vaccination.

### Laboratory Tests

A central laboratory performed hemagglutination-inhibition (HAI) and neutralizing (neut) antibody assays on all serum specimens as described [16], [17]. The starting dilution of serum for each assay was 1∶10. Another laboratory experienced with avian influenza virus serology tested a sample of the specimens. The primary study site also did HAI tests on the day 0 and 56 serum samples using a starting dilution of 1∶4 as described [18]. Planned analyses were for number of subjects developing a ≥4 fold increase, the number with a final titer ≥1∶40 and the geometric mean titer (GMT) for each group in the serum antibody tests.

### Analyses

#### Reactogenicity

The number of subjects in each vaccine group reporting a specific solicited local or systemic adverse effect (AE) that was mild, moderate or severe for seven days after each vaccination and the number and description of unsolicited AEs for 28 days after each vaccination were totaled. Occurrence of severe AEs (SAE) was monitored for six months and individually evaluated in detail for any potential vaccine relationship.

#### Immunogenicity

The number of subjects in each vaccine group developing a ≥4-fold serum antibody response and achieving a final titer of ≥1∶40 in HAI and neut assays was totaled 28 days after each vaccination and at 208 days. Also, the number (%) of subjects developing a ≥4-fold and ≥1∶32 final titer 28 days after dose 2 (day 56) in the more sensitive HAI assay was totaled. GMT calculations were not done.

#### Statistical Analyses

Statistical analyses were conducted using SPSS version 17.0.

### Registry Name, Number and Performance

#### Name

Safety, reactogenicity, and immunogenicity of inactivated influenza A/H7/N7 vaccine in healthy adults. No.: NCT00546585. Initiated March 10, 2008, completed February 10, 2009.

#### Performance

Study performance conformed to CONSORT guidelines and is reported conforming to Consort 10 guidelines (PLoS Medicine 2010, 7(3):1–7 e1000251).

## Results

### The Study

**Figure 1:**
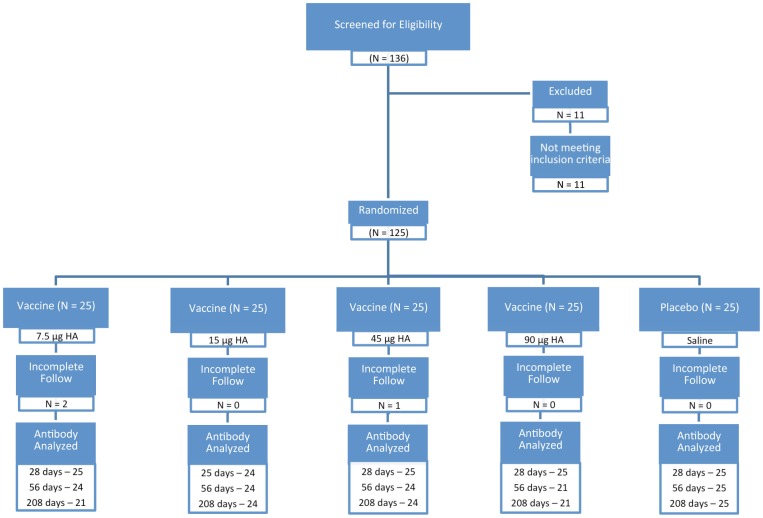
Flow diagram of the randomized clinical trial of an inactivated subunit influenza A (H7N7) vaccine.

A flow diagram of the randomized trial is shown in Figure 1. One hundred thirty-six subjects were screened and 125 were enrolled. Eleven persons were excluded, one for over age 40, four for a blood pressure exceeding 140/90, four for a new or changed medication within the three month limit, one for having received a recent vaccination, one for a positive pregnancy test and one for an unstable medical condition.

One hundred twenty-five subjects were randomized; complete followup for 208 days was available on 122 (97.6%). Of the three who withdrew, one thought dose 1 had caused an acute respiratory illness and two withdrew for travel. Four others were not given dose 2 because of an SAE (see reactogenicity), and one of these had started a new medicine (exclusion criterion). The number of subjects in each vaccination group tested for antibody at each specified time is shown in the figure.

Demographics of the 125 enrolled subjects are shown in Table 1. The median age was 26.4 years and the range was 18 to 40 years. The male and female percent of participants was 49 and 51%, respectively, and most subjects were white. There were no significant differences among the different dosage groups by gender, race, or age (Table 1).

**Table 1:** Demographics by Study Group.

	ALL (No. = 125)	7.5 mcg (No. = 25)	15 mcg (No. = 25)	45 mcg (No. = 25)	90 mcg (No. = 25)	Placebo (No. = 25)
**Gender – No. (%)** 1						
Male	61 (49)	10 (40)	11 (44)	14 (56)	13 (52)	13 (52)
Female	64 (51)	15 (60)	14 (56)	11 (44)	12 (48)	12 (48)
**Race – No. (%)** 2						
American Indian/Alaskan Native	0	0	0	0	0	0
Asian	14 (11)	4 (16)	2 (8)	2 (8)	4 (16)	2 (8)
Hawaiian/Pacific Islander	0	0	0	0	0	0
Black/African American	10 (8)	2 (8)	0	5 (20)	1 (4)	2 (8)
White	94 (75)	19 (76)	23 (92)	15 (60)	18 (72)	19 (76)
Multi-Racial	6 (5)	0	0	2 (8)	2 (8)	2 (8)
Other/Unknown	1 (1)	0	0	1 (4)	0	0
**Age in Years** 3						
Mean (STD)	28.1 (5.0)	29.1 (5.5)	29.6 (5.5)	27.8 (4.9)	28.2 (4.8)	25.7 (3.7)
Median	26.4	28.8	28.4	26.1	28.0	25.2
Min,Max	(18, 40)	(22, 40)	(18, 40)	(21, 38)	(19, 38)	(20, 38)

1 Male vs. nonmale frequency by dosage, chi-square 1.73, p = .785.

2 White vs. nonwhite frequency by dosage, chi-square 7.04, p = .134.

3 Age vs. dosage, Anova, p>.05.

### Reactogenicity

Solicited adverse events in the seven days following each vaccination are summarized in Table 2. After dose one, fever (≥37.7°C), was reported by one subject given the 7.5 µg dosage and two given the 90 µg dosage. After dose two, fever was reported by one subject given the 7.5 µg dosage and one given 45 µg. The most commonly reported systemic symptoms in the week postvaccination were headache and malaise; the most commonly reported local symptoms were redness and tenderness. There was an increased frequency of moderate AE among those given the higher dosages but there were no severe AEs. There were no severe unsolicited reactions; mild or moderate unsolicited AEs thought to be vaccine related were transient and were injection site bruise – 2, site pruritus – 1, dizzy – 2, vomiting – 1, throat irritation – 1, anxiety – 1, and a new herpes labialis lesion – 1. Overall, the vaccinations were well tolerated.

**Table 2:** Solicited Adverse Events (AE) after Influenza A (H7N7) Vaccinatons.

		No. (%) with ≥One AE in Category							
		Dose 1				Dose 2			
Adverse Events	Dosage^1^ (µg HA)	No.^2^	Mild	Mod	Severe	No.^2^	Mild	Mod	Severe
Systemic	0	25	5 (20)	3 (12)	0	22	5 (23)	1 (5)	0
	7.5	25	10 (40)	1 (4)	0	24	1 (4)	1 (4)	0
	15	25	9 (36)	2 (8)	0	24	5 (21)	1 (4)	0
	45	25	5 (20)	2 (8)	0	23	3 (13)	0 (0)	0
	90	25	4 (16)	6 (24)	0	25	4 (16)	1 (4)	0
Local	0	25	13 (52)	0 (0)	0	22	12 (55)	0 (0)	0
	7.5	25	15 (60)	0 (0)	0	24	14 (58)	0 (0)	0
	15	25	11 (44)	2 (8)	0	24	17 (71)	1 (4)	0
	45	25	14 (56)	1 (4)	0	23	15 (65)	3 (13)	0
	90	25	14 (56)	3 (12)	0	25	13 (52)	3 (12)	0

1 As determined in single radial immunodiffusion assays.

2 Number subjects evaluated.

Four subjects were designated as an SAE during the study period. Two persons were hospitalized, one for moderately severe back pain considered related to a car accident two years earlier but the hospitalization was extended because of depression; both illnesses resolved without sequelae. The other hospitalization was for stress-related severe depression that improved during hospitalization and was considered resolved on discharge. A 40-year old female was noted to have eyelid edema when reporting for dose two and reported a new physician-prescribed medication; she also reported experiencing eyelid edema in the past. Follow-up evaluations led to a diagnosis of mixed connective tissue disease and special tests on her prevaccination and earlier available sera revealed evidence of a preexisting autoimmune disorder. The illness was considered moderately-severe. None of these three subjects were given the second vaccination. Both the site investigators and the independent safety monitoring committee considered these SAEs as not associated with vaccination. The fourth SAE was a mild transient decrease in hearing by the left ear with onset nine days after the initial vaccination that did not interfere with normal activities. A specific cause other than vaccination was not identified and treatment provided by ENT consultation did not lead to improvement. Hearing returned to normal in 84 days; the second vaccination was not done. Both the site investigators and the independent safety monitoring committee designated the AE as vaccination associated; the independent monitoring committee required an SAE designation because of “significant disability.”

### Immunogenicity

Serum antibody response measurements at the central laboratory 28 days after each vaccination are shown in Table 3. As noted, very few significant responses were seen but most were after the 45 and 90 µg dosages. None of the subjects retained a 4-fold response and titer ≥1∶40 in HAI tests of sera collected at 208 days and only one subject (15 µg HA dosage) retained an increase and final ≥1∶40 titer in neut tests (data not shown). The infrequent responses reported by the central laboratory were confirmed by the laboratory with experience in serology for avian influenza viruses (data not shown). Results of testing for HAI antibody at the primary test site are shown in Table 4. The greater sensitivity of the assay revealed a dose response in frequency of significant increases in titer (logistic regression, p = .001) although the frequency for the highest dosage was only 36% and only two of the subjects achieved a titer of 1∶32.

**Table 3:** Serum Antibody Responses to Influenza A (H7N7) Vaccinations^1^.

	Post Dose 1					Post Dose 2				
		No. (%) ≥4-Fold Increase		No. (%) ≥1∶40			No. (%) ≥4-Fold Increase		No.(%) ≥1∶40	
Dosage (µg HA)^2^	No.^3^	HAI^4^	Neut^4^	HAI^4^	Neut^4^	No.^3^	HAI^4^	Neut^4^	HAI^4^	Neut^4^
0	25	0	0	0	0	25	0	0	0	0
7.5	25	0	0	0	0	24	0	0	0	0
15	24	0	1 (4)	0	1 (4)	24	0	1 (4)	0	1 (4)
45	25	0	0	0	0	24	0	2 (8)	0	2 (8)
90	25	0	0	0	0	21	1 (5)	2 (10)	1 (5)	2 (10)

1 As determined at central laboratory; increase defined as ≥4-fold or <10 to 40.

2 As determined in single radial immunodiffusion assays.

3 Number of subjects in evaluation.

4 Hemagglutination-inhibition (HAI) and neutralizing (neut) antibody assays at central laboratory.

**Table 4:** Serum Antibody Responses to H7N7 Vaccinations^1^.

		HAI Antibody Post Dose 2	
Dosage/µg HA^2^	No.^3^	No. (%) ≥4 Fold Increase^4^	No. (%) ≥1∶32
0	25	0	0
7.5	21	2 (9.5)	0
15	21	2 (9.5)	1 (5)
45	24	6 (25)	0
90	22	8 (36)	1 (5)

1 Hemagglutination-inhibition antibody assays at primary study 'site.

2 As determined in single redial immunodiffusion assays.

3 Number of subjects in evaluation.

4 Increasing dosage induced increased % increase; logistic regression, p = .001.

## Discussion

The major finding of this study is that an inactivated influenza A (H7N7) subunit vaccine prepared using manufacturing methods approved for seasonal influenza vaccines was poorly immunogenic in healthy young adults despite their having been given two doses a month apart of dosages up to 90 µg of the HA as determined in SRID assays. The neut assay at the central laboratory appeared to be slightly more sensitive than the HAI assay whereas the HAI assay appeared more sensitive than the neut assay at a laboratory with considerable experience with avian influenza virus serology (data not shown). Nevertheless, the serologic findings at the central laboratory were essentially confirmed. The more sensitive HAI assay performed at the study site provided a more complete assessment of immunogenicity. That test indicated that immunogenic HA was present in the vaccine although the immunogenicity was still considerably lower than what was expected for the dosages administered. The major limitation of these findings is that the sample size was not selected to produce definitive immunogenicity data on any of the dosages tested. However, the desired guidance for any further testing was obtained.

Influenza A (H7N7) is proposed as a priority for vaccine development for potential use in humans along with H5N1 and H9N2 because of human infections that have been detected with these viruses [3], [19]. The case for H7N7 was emphasized when an outbreak with H7N7 viruses occurred in 2003 in the Netherlands among persons exposed to poultry; one infected person died and evidence for human-to-human transmission was provided [9], [10]. Also, in an outbreak of H7N3 infections in poultry in Canada in 2004, transmission to humans was detected [20], [21].

Considerable effort has been expended on vaccine development with H5N1 viruses for potential use in humans [22]. Influenza A/H7 vaccines have been developed and shown to be immunogenic and to induce protection in animals [13], [23]–[25]. Thus, the H7 HA appears fully immunogenic in vaccinations of animals and should also be fully immunogenic for humans. Other clinical trials in humans with H7 vaccines have been reported. Low immunogenicity was reported in a clinical trial using a split virus vaccine prepared in tissue culture with A/H7N1 plasmids derived from a chicken virus [26]. Two vaccinations three weeks apart of 12 and 24 µg dosages of the HA with and without alum were each given to 15 healthy adults (total 60). The 12 and 24 µg dosages without alum induced HAI and/or neut antibody responses in 21 and 23% of subjects, respectively and 50 and 62% respectively with adjuvant. A clinical trial with an H7N3 live attenuated virus using the A/Ann Arbor (H2N2) cold-adapted Master Donor Strain given to 17 subjects showed minimal virus shedding but serum HAI and neut antibody responses in 43% and 48%, respectively [27].

The present report and the two summarized clinical trials, in combination with the good immunogenicity in animal immunizations, indicate that a satisfactory H7 vaccine can be made for humans. Because of the potential need for an H7N7 vaccine for humans, developing a vaccine for potential use in humans seems indicated. To aid in this goal, we have directed research toward gaining some understanding of why this H7N7 vaccine of high HA dosage was so poorly immunogenic in humans. These studies are reported in a companion report on in vitro correlates of immunogenicity of avian influenza virus vaccines in humans.

## Supporting Information

Checklist S1 **Consort checklist.**
(DOC)

Protocol S1 **Study protocol.**
(PDF)
