# Supplementary material for: A Randomized Clinical Trial of an Inactivated Avian Influenza A (H7N7) Vaccine
Source: PLoS One. 2012 Dec 11;7(12):e49704. doi: 10.1371/journal.pone.0049704 (PMC3519847; doi:10.1371/journal.pone.0049704)
Supplement: Protocol S1 — Study protocol. (PDF) [file pone.0049704.s002.pdf]

✓Compare

Institutional Review Board for Baylor College of Medicine and Affiliated Hospitals

Protocol Number: **H-21595**
Status: Approved
Initial Submit Date: 10/3/2007
Approval Period: 10/6/2009 - 8/17/2010

Section Aa: Title & PI

A1. Protocol Title

A RANDOMIZED, DOUBLE-BLINDED, PLACEBO-CONTROLLED, PHASE I/II, DOSE-RANGING STUDY OF THE SAFETY, REACTOGENICITY, AND IMMUNOGENICITY OF INTRAMUSCULAR INACTIVATED SUBVIRION INFLUENZA A/H7N7 VACCINE IN HEALTHY ADULTS. (DMID NO. 07-0023, VER. 3.0, 15 JAN 2008)

A2. Principal Investigator

Name:	ROBERT BARNARD COUCH	Phone:	713-798-4474
Id:	737632	Fax:	713-798-8344
Department:	MOLECULAR VIROLOGY & MICROBIOLOGY	Email:	rcouch@bcm.tmc.edu
Center:		Mail Stn:	BCM280

A3. Administrative Contact

Name:	I. DARLENE KIRK	Phone:	713-798-4474
Id:	029894	Fax:	713-798-8344
		Email:	ikirk@bcm.tmc.edu
		Mail Stn:	BCM280

A3a. Financial Conflict of Interest

Does the investigator have a financial interest in any non-Baylor sponsor or funding source for this research?
No

A3b. Cooperative Agreement

Is this a cooperative agreement protocol?
No

Which institution is the IRB of record?
BCM: Baylor College of Medicine

Section Ab: General Information

A4. Co-Investigators

Name:	WENDY A. KEITEL	Phone:	713-798-5250
Id:	016087	Fax:	713-798-6802
Department:	MOLECULAR VIROLOGY & MICROBIOLOGY	Email:	wkeitel@bcm.tmc.edu
Center:		Mail Stn:	BCM280

Name:	SHITAL MAHENDRA PATEL	Phone:	713-798-3793
Id:	144006	Fax:	713-798-8948
Department:	MEDICINE: INFECTIOUS DISEASE	Email:	shitalp@bcm.tmc.edu
Center:		Mail Stn:	BCM280
Name:	HANA MOHAMMED EL SAHLI	Phone:	713-798-2058
Id:	672312	Fax:	713-798-6802
Department:	MOLECULAR VIROLOGY & MICROBIOLOGY	Email:	hanae@bcm.tmc.edu
Center:		Mail Stn:	BCM280
Name:	ROBERT LEGARE ATMAR	Phone:	713-798-6849
Id:	719057	Fax:	713-798-6802
Department:	MEDICINE: INFECTIOUS DISEASE	Email:	ratmar@bcm.edu
Center:		Mail Stn:	BCM280

A5. Funding Source:

NIH

A6a. Institutions where work will be performed:

BCM: Baylor College of Medicine

A6b. Research will be conducted outside of the United States:

Country:
Facility/Institution:
Contact/Investigator:
Phone Number:

If documentation of assurances has not been sent to the Office of Research, please explain:

A7. Research Category:

Section B: Review Path Determination

B1. Full Board or Expedited Review

Is this an compassionate/emergency use situation?
No

If this is a drug study, is an investigational new drug (IND) application required?
N/A

If this is a device study, is an investigational device exemption (IDE) application required?
N/A

If the research involves ONLY blood collection, are subjects healthy, non-pregnant adults whose weight is at least 110 pounds, with amount drawn less than 550 ml in an 8 week period, and with collection not occurring more frequently than 2 times per week?
N/A

If the research involves ONLY blood collection for other adults and children, considering age, weight and health of subjects, is the amount drawn in an 8 week period less than 50ml or 3 ml per kg, and with collection not occurring more frequently than 2 times per week?
N/A

Does the research involve ONLY the collection of biological specimens for research purposes by noninvasive means?

(e.g. Hair; extracted teeth; excreta, sputum and external secretions; placenta removed at delivery; mucosal and skin cells collected by scraping or swab)

N/A

Does the research involve ONLY the collection of data through noninvasive procedures (not involving general anesthesia or sedation) routinely employed in clinical practice, excluding procedures involving x-rays or microwaves? (e.g. EKG, ECHO, EEG, Ultrasound, MRI)

N/A

Does the research involve ONLY materials (data, documents, records, or specimens) that have been collected, or will be collected solely for non-research purposes (such as medical treatment or diagnosis)?

N/A

Does the research involve ONLY the collection of data from voice, video, digital, or image recordings made for research purposes?

N/A

Does the research involve ONLY individual or group characteristics or behavior (including, but not limited to, research on perception, cognition, motivation, identity, language, communication, cultural beliefs or practices, and social behavior) or research employing survey, interview, oral history, focus group, program evaluation, human factors evaluation, or quality assurance methodologies?

N/A

Does the research involve pedigree studies, collection and/or storage of specimens for DNA analysis or gene transfer?

No

B2. Exempt From IRB Review

Not Applicable

B3. Waiver of Subject Authorization

Not Applicable

Section C: Background

Several influenza pandemics have been documented during the past century, including Spanish Flu (A/H1N1) of 1918, Asian Flu (A/H2N2) of 1957 and Hong Kong Flu (A/H3N2) of 1968. The most significant of these was Spanish Flu (A/H1N1) of 1918 with an estimate of up to 60 million deaths worldwide.

Although many years have elapsed since the last pandemic, the serious threat of pandemic influenza remains. One cause of concern for a new pandemic is "H5" bird flu that is presently active in Asia. An outbreak of another bird flu ("H7") occurred in the Netherlands in 2003; it made 83 people sick and one person died.

Availability of sufficient quantities of an appropriate vaccine could save thousands of lives and billions of dollars in the face of an influenza pandemic. The making and testing of H5N1, H7N7 and other investigational influenza vaccines during interpandemic periods are critical steps for protecting the the world's populations against these potential ravages. This protocol is concerned with initial testing in humans of Monovalent Influenza A/H7N7 Subvirion Vaccine against one of these potential pandemic strains. This vaccine was produced under contract to the US Dept. of Health and Human Services by Sanofi Pasteur Inc. in the company's Swiftwater, PA, facility employing current Good Manufacturing Practices (cGMP) and a predominantly pilot-scale process nearly identical to that of "Fluzone", the company's current US-licensed influenza vaccine. However, the A/H7N7 vaccine itself has undergone no preclinical pharmacology, toxicology, pharmacokinetic, or metabolism studies.

The investigational vaccine is based on an influenza A/H7N7 reference strain derived by classical genetic reassortment techniques to have its H7 and N7 from low pathogenic avian influenza viruses, its NP gene from A/Johannesburg /82/96 (H1N1), and the remaining five internal genes from A/Puerto Rico/8/34; this virus should be avirulent for animals and humans, and, furthermore, vaccine prepared from it is provided as a non-infectious subvirion product.

References: 1) Hampson AW and Mackenzie JS. Preparing for an Influenza Epidemic; The Influenza Viruses. Medical Journal of Australia 2006; 185:S39-S43. 2) Whitley RJ, Monto AS, Bartlett J, Hayden FG, Tapper M and Pavia AT,

Guest Editors. Seasonal and Pandemic Influenza: At the Crossroads, a Global Opportunity. J Infect Dis 2006; 194 (Suppl 2):S65-S161. 3) Du Ry van Beest Holle M, Meijer A, Koopmans M, de Jager CM, et al. Human-to-Human Transmission of Avian Influenza A/H7N7, The Netherlands, 2003. Eurosurveillance 2005; 10:264-8.

Section D: Purpose and Objectives

The goals of this study are to compare the safety, reactogenicity, and immungenicity of increasing doses of mononvalent subvirion influenza A/H7N7 virus vaccine administered as primary and booster intramuscular (deltoid) injections four weeks apart to healthy adults 18 through 40 years of age. The A/H7N7 vaccine will be supplied in 1.0 ml, single-dose vials containing 15, 30 and 90 micrograms (mcg) HA per ml and no antibiotics or preservatives; doses of 7.5, 15 and 45 mcg will be obtained by withdrawing only 0.5 ml from the sequential vials. A fourth vaccine dose, 90 mcg in 1.0 ml (ie, the complete contents of the 90 mcg/ml vial), and a Placebo, 0.5 ml of Normal Saline for Injection, will also be employed. The primary goal is to determine a dose of A/H7N7 vaccine that results in post-vaccination antibody titers that are likely to be protective along with an acceptable safety profile. Results from this trial may also serve as a guide for possible expanded phase II clinical trials.

Section E: Protocol Risks/Subjects

E1. Risk Category

(45 CFR 46.404) Category 1: Research not involving greater than minimum risk.

E2. Subjects

Gender:
Both

Age:
Adult (18-64 yrs)

Ethnicity:
All Ethnicities

Primary Language:
English

Groups to be recruited will include:
Healthy, non-patient, normals

Vulnerable populations to be recruited as subjects:
Employees or lab personnel, Students

Vulnerable populations require special protections. How will you obtain informed consent, protect subject confidentiality, and prevent undue coercion?

Notices concerning this study will be posted about the Texas Medical Center and thus may come to the attention of students, employees and/or lab personnel. However, none of these subjects will be sought out for recruitment; explanation of the study will only be offered upon their request. (Note that the age range will be 18 through 40 years rather than 18 through 64.) Serum will be transferred to an NIAID-designated laboratory under code. The code is maintained in a secure database that has restricted access.

E3. Pregnant woman/fetus

Will pregnant women be enrolled in the research?
No

E4. Neonates

Will neonates be enrolled in the research?

No

E5. Children

Will children be enrolled in the research?

No

Section F: Design/Procedure

F1. Design

Select one category that most adequately describes your research:

z.z) ARCHIVED DO NOT USE - Other: Drug, Phase I/II

Discuss the research design including but not limited to such issues as: probability of group assignment, potential for subject to be randomized to placebo group, use of control subjects, etc.

This influenza vaccine study will include four different vaccine doses, three containing 7.5, 15 or 45 mcg of H7 HA in 0.5 ml and one containing 90 mcg of H7 HA in 1.0 ml, plus normal saline placebo injections in a 0.5 ml volume. The total number of subjects falling into each of the five vaccine/placebo groups will be 25, thus giving each subject a 1 in 5 chance of being assigned to a particular group.

Inclusion Criteria:

1. Male or nonpregnant (negative urine pregnancy test required immediately prior to vaccine administration) and non-lactating female18 through 40 years of age. 2. Women of childbearing potential (not surgically sterile or postmenopausal for =/>1 year) must agree to practice adequate contraception (ie, barrier method, abstinence, intrauterine device, and/or a licensed hormonal method) for the entire study period. 3. Are in good health as determined by vital signs, medical history, and a targeted physical examination based on medical history. 4. Are able to understand and comply with planned study procedures. 5. Provide informed consent prior to initiation of any study procedures and are available for all study visits.

Exclusion Criteria:

1. Known allergy to eggs or other components of the vaccine icluding gelatin, formaldehyde, octoxinol (Triton 100), and chicken protein. 2. Positive urine or serum pregnancy test, or breast feeding. 3. Immunosuppression as a result of underlying illness or treatment, or use of cancer chemotherapy or radiation therapy within the preceeding 36 months. 4. Active neoplastic disease or a history of any hematologic malignancy. 5. Long-term use of oral steroids, parenteral steroids, or high-dose inhaled steroids (>800 mcg/day of beclomethasone dipropionate or equivalent) within the preceding 6 months (topical steroids are allowed). 6. Receipt of immunoglobulin or any other blood product within 3 months prior to enrollment in this study. 7. Receipt of any other licensed, inactivated vaccine within the preceding 2 weeks, or live vaccine within 4 weeks prior to vaccination in this study. 8. Presence of an acute or chronic medical condition that the investigator believes would render vaccination unsafe or would interfere with the evaluation of responses; examples include but are not limited to: chronic liver disease, renal insufficiency, unstable neurological disorders, diabetes mellitus, and transplant recipients. 9. History of a severe reaction to contemporary influenza virus vaccine. 10. Current or recent acute illness with an oral temperature greater than 100.4 degrees F within 1 week of vaccination. 11. Receipt of an experimental agent within one month prior to vaccination in this study, or planned within the 7 month study period. 12. Any condition that would, in the opinion of the site investigator, place the subject at an unacceptable risk of injury or render the subject unable to meet the requirements of the protocol. 13. Diagnosis of schizophrenia, bipolar disease, or other major psychiatric diagnosis. 14. Hospitalization for psychiatric illness, history of a suicide attempt, or confinement for danger to self or others. 15. Receiving psychiatric drugs, but may enroll if receiving a single antidepressant and stable for at least 3 months with no decompensating symptoms. 16. Active infection with HIV, HBV and/or HCV. 17. History of drug or alcohol abuse in the past 5 years. 18. Planned travel outside the USA between the first (day 0) and third (approximately day 56) blood samples. 19. History of Guillain-Barre syndrome. 20. Any condition that the investigator believes may interfere with successful completion of the study.

F2. Procedure

Screening and Enrollment Day 0, Visit 1 •The studies purpose and procedures will be described to potential subjects. They will be asked to read the consent form and obtain answers to any questions before signing it. •Eligibility criteria will then be reviewed, and temperature, pulse, and blood pressure measured. •A urine pregnancy test for all females with childbearing potential must be negative prior to vaccination. •Medical history will be reviewed and all concomitant

medications will be recorded. •A targeted physical examination may be performed, as indicated, and 20-mL venous blood sample for antibody assays will be collected. •Subjects will be enrolled in IDES to be stratified according to prior receipt of influenza vaccine and assigned randomly to a dose group. •Subjects will receive a single dose of vaccine (volume is dependent upon dose group) via IM injection in the deltoid muscle of the preferred arm and will be observed in the clinic for a minimum of 30 minutes following vaccination. The vaccination site will be examined, and any AEs will be assessed prior to discharge from the clinic. •Subjects will be provided with a memory aid and other study related materials to record daily oral temperature and systemic and local AEs. They will be asked to take their temperature around the same time each day, and instructed on how to use the memory aid and how to rate any AEs prior to discharge from the clinic. Subjects will be instructed to notify the study center if they develop any severe reactions following vaccination. If the investigator deems the reaction severe enough, he/she will give further instructions on the proper course of action, including a return to the clinic for immediate evaluation if appropriate. Subject Follow-Up Day 2, Visit 2 (window: Days 1 to 3) •Study personnel will contact the subject by telephone to solicit any AE and concomitant medication information, review information on the memory aid, and remind the subject to complete the information on the memory aid. Day 8, Visit 3 (window: Days 8 to 12) •Review current health status and note any changes since the last visit. •A targeted physical examination may be performed, as indicated. •Study personnel will collect and review the memory aid information with the subject and record all AEs and concomitant medications on the appropriate case report form (CRF). •A Safety Monitoring Committee review of available data by about day 20 before proceeding to administer second vaccine doses to any subjects. Day 28, Visit 4 (window: Days 26 to 30) •Eligibility criteria will be reviewed. Review current health status and note any changes since the last visit. •Vital signs will be collected, including temperature, pulse, and blood pressure. •All concomitant medications will be recorded. •All AEs will be recorded. •A urine pregnancy test will be performed for all female subjects of childbearing potential. Results must be negative prior to vaccination. •A targeted physical examination may be performed, as indicated. •A 20-mL venous blood sample for antibody assays will be collected prior to vaccination. •Subjects will receive a single dose of vaccine (volume is dependent upon dose group) via IM injection in the deltoid muscle of the preferred arm. Subjects will be observed in the clinic for a minimum of 30 minutes following vaccination. The vaccination site will be examined and any adverse events will be assessed prior to discharge from the clinic. •Subjects will be provided with a memory aid and other study related materials to record daily oral temperature, and systemic and local AEs. Subjects will be encouraged to take their temperature around the same time each day. Subjects will be instructed on how to use the memory aid and how to rate any adverse events prior to discharge from the clinic. Subjects will be instructed to notify the study center if they develop any severe reactions following vaccination. Day 30, Visit 5 (window: 1-3 days after receipt of second dose of vaccine) •Study personnel will contact the subject by telephone to solicit any AE and concomitant medication information, review information on the memory aid and remind the subject to complete the information on the memory aid. Day 36, Visit 6 (window: 8-12 days after receipt of second dose of vaccine) •Review current health status and note any changes since the last visit. •A targeted physical examination may be performed, as indicated. •Study personnel will collect and review the memory aid information with the subject and record all concomitant medications and AEs on the appropriate CRF. Day 56, Visit 7 (window: 26-30 days after receipt of second dose of vaccine) •Review current health status and note any changes since the last visit. •All concomitant medications will be recorded. •All AEs will be recorded. •A targeted physical examination may be performed, as indicated. •A 20-mL venous blood sample for antibody assays will be collected. Day 208, Visit 8/Final Study Visit: (window 208-236 days after receipt of first dose of vaccine) •A 20-mL venous blood sample for antibody assays will be collected. •A targeted physical examination may be performed, as indicated. •Information regarding SAEs will be solicited. Any ongoing related SAEs will be followed to resolution or until a stable chronic condition has been established. •All concomitant medications will be recorded. Early Termination Visit •Review current health status since the last visit. •All concomitant medications will be recorded. •A 20-mL venous blood sample for antibody assays will be collected. •A targeted physical examination may be performed, as indicated. •Information regarding AEs will be solicited. Any ongoing related AEs will be followed to resolution or until a stable chronic condition has been established. Subjects will be encouraged to permit continued follow-up of AEs and to donate scheduled blood samples, if possible.

Section G: Sample Size/Data Analysis

G1. Sample Size

How many subjects (or specimens, or charts) will be used in this study?

Local: 125 Worldwide: 125

Please indicate why you chose the sample size proposed:

The number of recipients of the four different vaccine strengths in this pilot study were chosen by the sponsors to provide an initial assessment of the vaccine's immunogenicity and reactogenicity, and to serve as a guide for further studies with the vaccine. The choice to add a similar number of placebo recipients was initiated by the present investigators.

G2. Data Analysis

Provide a description of your plan for data analysis. State the types of comparisons you plan (e.g. comparison of means, comparison of proportions, regressions, analysis of variance). Which is the PRIMARY comparison/analysis? How will the analyses proposed relate to the primary purposes of your study?

For analyses to be performed by the NIH contractor, we will provide reports of reactogenicity of the different vaccine strengths and placebo, and serum samples from the volunteers .

Section H: Potential Risks/Discomforts

H1. Potential Risks/Discomforts

Describe and assess any potential risks/discomforts and assess the likelihood and seriousness of such risks:

The discomforts of this study include having blood drawn, intramuscular (IM) injection of the vaccine, and possible reactions to the vaccine. Drawing blood causes transient discomfort and may cause fainting. Bruising at the blood draw site may occur but can be prevented or lessened by applying pressure to the draw site for several minutes. Intramuscular injection also may cause transient discomfort. The use of alcohol swabbing and sterile equipment will make infection at the site where blood will be drawn or where the vaccination is given extremely unlikely. Reactions to the A/H7N7 vaccine are expected to be similar to those that occur with regular annual influenza vaccinations, and may include influenza-like reactions such as fever, body aches, headache, malaise, myalgia, and/or nausea. These may occur more frequently in people who are given the higher dose levels of vaccine. These reactions are usually greatest within the first 24 hours after vaccination and last 1 to 2 days. Some subjects may develop reactions at the site of vaccination (redness, swelling, pain, or tenderness), and individuals in this study who receive the higher vaccine dose levels have a greater likelihood of developing these reactions. Analgesics (e.g., ibuprofen or acetaminophen) and rest will generally relieve or moderate these symptoms. These reactions should go away in 1 to 4 days and should not require additional treatment. Acute and potentially life-threatening allergic reactions are also possible. During the swine influenza vaccine campaign of 1976, about 1 per 100,000 vaccine recipients developed a paralytic illness called Guillain-Barré Syndrome. This has not been seen consistently with other influenza vaccines. Most persons who develop Guillain-Barré Syndrome recover completely. There may be other unknown side effects.

H2. Data and safety monitoring plan

Do the study activities impart greater than minimal risk to subjects?

No

H3. Coordination of information among sites for multi-site research

Is the BCM Principal Investigator acting as the SPONSOR-INVESTIGATOR for this multi-site research?

No or Not Applicable

Is BCM the COORDINATING CENTER for this multi-site research?

No or Not Applicable

Section I: Potential Benefits

Describe potential benefits to be gained by the individual subject as a result of participating in the planned work.

Potential direct benefits of this study for you might occur if you were exposed to the "H7" influenza in a farm setting or an outbreak of "H7" bird flu infections occurred, and the study vaccine induced protective antibodies against this virus. However, the study vaccine will not protect against influenza viruses that regularly circulate annually, nor against the H5N1 avian influenza virus that is causing sporadic and often fatal disease elsewhere in the world. Moreover, one of every five subjects in this study will receive an injection of plain sterile saline, reactions to which will serve as a baseline for those to the vaccine. Thus, a direct protective effect from the study injection is very unlikely.

Describe potential benefits to society of the planned work.

Your participation should benefit society by increasing our understanding of how to best prepare vaccine for a future influenza pandemic, and the quantities of a novel influenza virus antigen that may be required to induce levels of antibody that are expected to be protective.

Do anticipated benefits outweigh potential risks? Discuss the risk-to-benefit ratio.

The knowledge gained about vaccine for a potential pandemic strain of influenza could be highly valuable if an outbreak of infection with such a virus erupted; this benefit is felt to significantly exceed the risk of a reaction to the vaccine.

Section J: Consent Procedures

J1. Waiver of Consent

Will this research require a waiver of consent and authorization?

No

Will additional pertinent information be provided to subjects after participation?

No

Explain why providing subjects additional pertinent information after participation is not appropriate.

Information of possible interest might include whether vaccine or placebo was received, the dose of vaccine that the subject received, and the subject's antibody response to the vaccine. Information about the preparation received could be supplied by the unblinded vaccinater if needed for evaluating a possible vaccine reaction, but it is otherwise desirable to maintain blinding until all evaluations are complete. Since antibody responses will be performed elsewhere, it is unclear whether or when that information may be available for possible transmission to subjects, and this information lacks pertinence in the absence of an outbreak of infection with an H7N7 influenza virus.

J1a. Waiver of requirement for written documentation of Consent

Is this research subject to FDA regulations?

Yes

Explain how the research involves no more than minimal risk to the participants, and the specifics demonstrating that the research does not involve procedures for which written consent is normally required outside of the research context.

N/A

J2. Consent Procedures

Who will recruit subjects for this study?

PI

PI's staff

Describe how research population will be identified, recruitment procedures, and consent procedures in detail.

The primary means of recruitment will be notices placed around the Texas Medical Center, and mailings to subjects who participated in prior studies and expressed an interest in being notified about future studies. Potential subjects who come to the study site will be given a Consent Form and a brief explanation of the study. After they have read the Consent Form and received answers to any questions they may have, they may chose to sign the form. After the form is signed, the subject's eligibility will be reviewed and, if satisfactory, they will be enrolled.

Are foreign language consent forms required for this protocol?

No

J3. Privacy and Intrusiveness

Will the research involve observation or intrusion in situations where the subjects would normally have an expectation of privacy?

No

J4. Children

Will children be enrolled in the research?

No

J5. Neonates

Will non-viable neonates or neonates of uncertain viability be involved in research?

No

J6. Consent Capacity - Adults who lack capacity

Will Adult subjects who lack the capacity to give informed consent be enrolled in the research?

No

J7. Prisoners

Will Prisoners be enrolled in the research?

No

Section K: Confidentiality

Will research data include health information by which subjects can be identified?

Yes

Where will research data be kept? How will such data be secured?

Research data available will be kept in secure locations with restricted access.

Who, besides the PI, the study staff, the IRB and the sponsor, will have access to identifiable research data?

No one.

Will you obtain a Certificate of Confidentiality for this study?

No

Please further discuss any potential confidentiality issues related to this study.

None known.

Section L: Cost/Payment

Delineate clinical procedures from research procedures. Will subject's insurance (or subject) be responsible for research related costs? If so state for which items subject's insurance (or subject) will be responsible (surgery, device, drugs, etc). If appropriate, discuss the availability of financial counseling.

The clinical procedures involved in this research are pregnancy tests on females prior to both vaccinations at time 0 and approximately 1 month; four blood draws of approximately (20 mL or 4 teaspoonfuls each; 16 teaspoonfuls total) from an arm vein at approximately 0, 1, 2 and 7 months; measurements of oral temperature, checking for local reations to the primary and booster intramuscular vaccinations in the upper arm, and targeted physical examinations to evaluate intercurrent complaints. Costs of these procedures are covered as part of the research.

If an injury that requires additional medical care occurs because of this study, the volunteer will receive that care but they or their insurance will have to pay for it just like any other medical care. Subjects will not be paid for the injury.

If subjects will be paid (money, gift certificates, coupons, etc.) to participate in this research project, please note the total dollar amount (or dollar value amount) and distribution plan (one payment, pro-rated payment, paid upon completion, etc) of the payment.

Dollar Amount:

250

Distribution Plan:

For the study with no extra visits, $200 will be paid at the 2 month visit, and $50 at the final visit about 7 months after the first vaccination. If the investigator requests extra visits for a reactogenicity, blood safety, or immunogenicity test, a payment of $25 will be added for each visit and $50 if blood is obtained.

Section M: Genetics

How would you classify your genetic study?

Discuss the potential for psychological, social, and/or physical harm subsequent to participation in this research. Please discuss, considering the following areas: risks to privacy, confidentiality, insurability, employability, immigration status, paternity status, educational opportunities, or social stigma.

N/A

Will subjects be offered any type of genetic education or counseling, and if so, who will provide the education or counseling and under what conditions will it be provided? If there is the possibility that a family's pedigree will be presented or published, please describe how you will protect family member's confidentiality?

N/A

Section N: Sample Collection

SAMPLE: Blood

What is the purpose of the sample collection?

Blood samples collected from an arm vein before each vaccination (days 0 and approximately 28), and approximately 1 and 6 months after the second vaccination will be used to determine the antibody response to the vaccine.

For blood draws, specify the amount drawn, in teaspoons, at each visit and across the course of the subjects entire participation time.

The amount of blood collected will be 20 ml (4 teaspoons) at each of the four blood draws, with the total being 80 ml (16 teaspoons).

Is there the possibility that cell lines will be developed with this sample?No

Sample will be obtained from:

Will the sample be stripped of identifiers?

No

If sample will be released outside the hospital:

Will sample be released to anyone not listed as an investigator on the protocol? Will the information be identifiable, coded or de-identified?

Coded samples will be analyzed for antibody response to the vaccine by an outside laboratory.

Will sample material be sold or transferred to any third parties? Will the information be de-identified?

No samples will be sold, but transfer to sponsor-designated person in coded form is possible.

If sample will be banked for future use:

Where will the sample be banked and for how long?

Coded residual serum samples will be retained at BCM and/or at a site designated by the sponsors at NIH. Study records required to identify donors of the coded samples will be in secure, confidential study records. The duration of retention is not known. There are no specific plans for future testing; however, a potential value of these samples would be in documenting whether H7N7antibodies induced by the vaccine exhibit good reactivity with some future H7N7 strain that exhibits epidemic potential.

Does the banking institution have an approved policy for the distribution of samples?

Yes.

If the entire sample will NOT be used during the course of this research study:

Will the remaining tissue be discarded? If not what will be done with the remaining sample after study completion and how long will the sample be kept?

The duration of retention is indefinite.

Will samples be made available to the research subject (or his/her medical doctor) for other testing?

No

If a subject withdraws from the study:

Will subject have the option to get the remaining portion of their sample back?
No

Will samples be destroyed? If not, will they be kept anonymously? What will happen to the sample if the subject revokes authorization?
Retained samples will be coded and will require confidential study records to break the code. If a subject revokes permission to retain his/her serum sample(s), they will be autoclaved and discarded.

Will data obtained from their sample be deleted? What will happen to the sample if the subject revokes authorization?
Annonymous data obtained from the sample will not be deleted. If a subject revokes permission to retain his/her serum sample(s), they will be autoclaved and discarded.

Will study data or test results be recorded in the subject's medical records?
No

Will results of specific tests and/or results of the overall study be revealed to the research subject and or his/her doctor?
Results of the overall study, when available, will be revealed to the research subject upon request.

Please identify all third parties, including the subject's physician, to receive the test results.
Individual test results will not be supplied to the subject, his/her physician, or any third parties. The sponsor (NIH) will receive results.

Section O: Drug Studies

Is this study placebo-controlled?
Yes

Does the research involve a drug or biologic (including radioactive drugs) that is not approved by the FDA?
Yes

Will the research involve a radioactive drug that is not approved by the FDA?
No

IND Number:
13517

Section P: Device Studies

Does this research study involve the use of ANY device?
No

Section Q. Consent Form(s)

None

Section R: Advertisements

ADVERTISEMENT: BCM Clinical Trials Website

Exact language of Advertisement:
Advertisement is attached in Section S dated 18 March 2008.
